# Importance of Germline and Somatic Alterations in Human *MRE11*, *RAD50*, and *NBN* Genes Coding for MRN Complex

**DOI:** 10.3390/ijms24065612

**Published:** 2023-03-15

**Authors:** Barbora Otahalova, Zuzana Volkova, Jana Soukupova, Petra Kleiblova, Marketa Janatova, Michal Vocka, Libor Macurek, Zdenek Kleibl

**Affiliations:** 1 Institute of Medical Biochemistry and Laboratory Diagnostics, First Faculty of Medicine, Charles University and General University Hospital in Prague, 12800 Prague, Czech Republic; 2 Department of Biochemistry, Faculty of Natural Science, Charles University in Prague, 12800 Prague, Czech Republic; 3 Institute of Biology and Medical Genetics, First Faculty of Medicine, Charles University and General University Hospital in Prague, 12800 Prague, Czech Republic; 4 Department of Oncology, First Faculty of Medicine, Charles University and General University Hospital in Prague, 12800 Prague, Czech Republic; 5 Laboratory of Cancer Cell Biology, Institute of Molecular Genetics, Czech Academy of Sciences, 14220 Prague, Czech Republic; 6 Institute of Pathological Physiology, First Faculty of Medicine and General University Hospital in Prague, 12853 Prague, Czech Republic

**Keywords:** NBN, MRE11, RAD50, NBS, ATLD, NBSLD, TP53, DNA repair, hereditary cancer syndromes, variant interpretation, NGS

## Abstract

The *MRE11*, *RAD50*, and *NBN* genes encode for the nuclear MRN protein complex, which senses the DNA double strand breaks and initiates the DNA repair. The MRN complex also participates in the activation of ATM kinase, which coordinates DNA repair with the p53-dependent cell cycle checkpoint arrest. Carriers of homozygous germline pathogenic variants in the MRN complex genes or compound heterozygotes develop phenotypically distinct rare autosomal recessive syndromes characterized by chromosomal instability and neurological symptoms. Heterozygous germline alterations in the MRN complex genes have been associated with a poorly-specified predisposition to various cancer types. Somatic alterations in the MRN complex genes may represent valuable predictive and prognostic biomarkers in cancer patients. MRN complex genes have been targeted in several next-generation sequencing panels for cancer and neurological disorders, but interpretation of the identified alterations is challenging due to the complexity of MRN complex function in the DNA damage response. In this review, we outline the structural characteristics of the MRE11, RAD50 and NBN proteins, the assembly and functions of the MRN complex from the perspective of clinical interpretation of germline and somatic alterations in the *MRE11*, *RAD50* and *NBN* genes.

## 1. Introduction

Maintenance of the tissue homeostasis relies on intracellular pathways regulating the genome stability, DNA integrity, and appropriate immune surveillance. Although DNA is a chemically stable molecule, its integrity is continually threatened by various endogenous or exogenous processes that alter the structural organization of DNA at different levels, from bases to nucleoprotein assembly of chromatin [1]. Elimination of DNA lesions is mediated by specific DNA repair pathways. Their activation is carefully integrated into a complex intracellular signaling network called the DNA damage response (DDR) [2]. Large number of proteins encoded predominantly by tumor suppressor genes are involved in DNA repair and the DDR. Their biallelic pathogenic variants are responsible for certain rare recessive syndromes, monoallelic germline alterations cause hereditary cancer predisposition, and somatic loss-of-function variants contribute to the development of sporadic tumors.

Deliberate formation of DNA double-strand breaks (DSB) occurs physiologically during meiotic chromosome recombination in gametes and V(D)J recombination in lymphocytes, ensuring the desired genome plasticity [3,4] (not covered in this review). Most commonly, DSB arise as toxic DNA lesions with the highest tumor-promoting potential in mitotically active cells [5]. In these pathological settings, the DSB result from ionizing radiation exposure, genotoxic DNA impairment caused by various intrinsic processes, or exogenous chemicals that threatened DNA (referred to as clastogens) [3]. The DNA DSB during cell division compromises the symmetric distribution of the replicated DNA into daughter cells, leading to genome rearrangements affecting many genes. The DSB are predominantly repaired by two different pathways, which include homology-directed repair or non-homologous end-joining. The homologous recombination (HR) represents a precise but highly complex repair strategy that requires large number of proteins and sister chromatid as a template for DNA repair, limiting HR to the S and G2 phases of the cell cycle. HR is characterized by extensive processing of the broken DNA ends generating long 3′-ssDNA overhangs that invade the sister chromatid to search for homologous template. In contrast, non-homologous end joining (NHEJ) utilizes only a limited set of proteins (including the Ku proteins serving as markers of canonical NHEJ pathway) that stabilize the broken DNA ends that are immediately re-ligated by the ligase IV complex. Fast NHEJ is a dominant mode of DSB repair in human cells; however, processing of DNA ends during the NHEJ can introduce a DNA sequence errors with functional consequences when DSB occur in coding or regulatory gene regions. Microhomology-mediated end joining (MMEJ, also termed alternative non-homologous end joining (Alt-NHEJ)) is a recently described repair pathway that depends on resection of DNA ends. However, instead of using the sister chromatid as a template, MMEJ ligates the DNA ends in the microhomology regions causing DNA deletions contributing to chromosome rearrangements [6].

DSB repair is initiated by the MRN complex consisting of three highly conserved nuclear proteins, MRE11, RAD50, and NBN. The MRN complex serves as a central hub that senses, processes, and signals DSB and directs the repair strategy to HR or NHEJ through interactions with proteins processing of the broken DNA ends [1,7,8]. The downstream MRN complex effectors in DDR include ATM (ataxia-telangiectasia mutated protein) and ATR (ataxia-telangiectasia and Rad3-related protein) kinases [9,10]. Alongside the participation in DNA repair, the MRN complex is involved in DNA replication in the S-phase, promotes degradation of the stalled replication forks, promotes telomere maintenance, cleans the DNA ends, ensures initial DNA resection, and prevents the DNA re-replication and senescence in mitotically active cells [11,12]. Thus, the prominent role of MRN complex in the maintenance of chromosome stability underscores the importance of germline (i.e., hereditary) and somatic (i.e., acquired) alterations in the genes coding for its structural components.

In this review, we describe the structural and functional characteristics of MRE11, RAD50, and NBN proteins, together with a brief description of the assembly and functional dynamics of the MRN complex. Furthermore, we describe the characteristics of pathological conditions caused by the presence of pathogenic alterations in the MRN complex genes. We reasoned that linking the structural, functional, and clinical information could be useful for the assessment of genetic alterations in the MRN complex genes identified by many next-generation sequencing (NGS) panels in patients with cancer or congenital neurological disorders.

## 2. Structure of the MRN Complex

**Figure 1:**
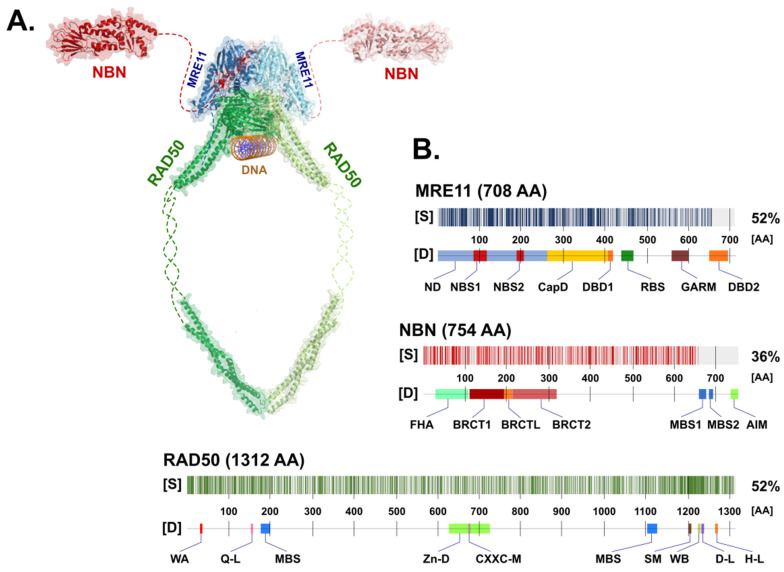
(**A**) Schematic appearance of the MRN complex interacting with dsDNA. Individual components include NBN (Protein Data Bank (PDB) ID: 3HUE; *Schizosaccharomyces pombe*), MRE11 (PDB ID: 4FBW; *S. pombe*), and RAD50 (PDB ID: 5DAC; *Chaetomium thermophilum*), modelled using PyMol (https://pymol.org/; version 2.5.2). Missing (non-crystallized) parts of the proposed structures for the NBN and RAD50 proteins are indicated by dashed lines. (**B**) The degree of similarity [S] between human (shown) and non-human paralogs of MRN complex proteins with the determined 3D structure used in panel (**A**). The positions of protein domains [D] of each MRN protein are shown in the figure. Abbreviations of the protein domains are explained in the text.

The MRN complex consists of a symmetrical dimer assembled from two MRE11, RAD50 protomers (M_2_R_2_) that are stabilized by NBN protein(s) [13]. The entire complex is a dynamic molecular structure consisting of a globular DNA binding domain and two coiled-coil arms protruding 60 nm apart [14]. Although the detailed molecular structure of the human MRN complex has not yet been solved, its structure should be similar in other species (Figure 1) due to the high conservation of structural and functional features of the MRN complex [15,16,17,18].

### 2.1. The Nuclease MRE11

**Figure 2:**
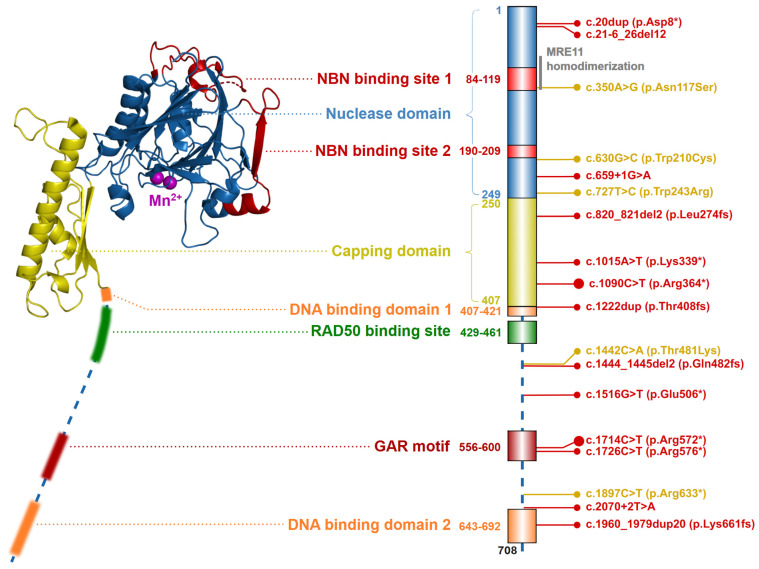
Schematic structure of the MRE11 protein (left; from *S. pombe*, PDB ID: 4FBQ). Two Mn^2+^ ions are shown as purple spheres in the center of the nuclease domain. The structure of flexible C-terminal parts (indicated by dashed line) containing the DNA binding domains, GAR motif, and RAD50 binding site were not resolved yet. Colors of protein domains in structure model (left) corresponds to that in schematic bar chart representing domain composition and the most frequent germline loss-of-function variants with allele frequency >10^−5^ in the GnomAD database (the size of the lollipop reflects the frequency of a variant). Yellow lollipops correspond to ATLD-causing variants. Grey notes highlight the important domain interactions.

The *MRE11* gene, localized on chromosome 11q21, codes for a canonical transcript consisting of 20 exons (19 coding; NM_005591). Its protein product, MRE11 nuclease (meiotic recombination 11 homolog; OMIM: *600814), consists of 708 amino acids (aa) forming an 81 kDa nuclear protein (Figure 2) [19].

Two MRE11 proteins homodimerize through their N-terminal nuclease domains (ND; aa residues 1–249). Each ND includes two NBN binding sites (NBS1; aa residues 84–119 and NBS2; aa residues 190–209) and five conserved phosphodiesterase motifs, that consist of five histidine residues (His22, His63, His129, His217, His245, His247), two aspartic acid residues (Asp20, Asp60) and an asparagine (Asn128), forming together a nuclease active site. The nuclease active site is bound by two Mn^2+^ ions which are essential for the ssDNA endonuclease and dsDNA exonuclease activities of MRE11. The first Mn^2+^ ion is bound to Asp20, His22, Asp60 and His247 and the second Mn^2+^ ion coordinated to Asp60, Asn128, His217 and His245 [20,21,22,23]. The adjacent capping domain (CapD; aa residues 230–406) helps to discriminate between ssDNA and dsDNA substrates and controls their correct entry into the nuclease active site [15,20,24]. Two DNA binding domains (DBD1; aa residues 407–421 and DBD2; aa residues 643–692) are located downstream of CapD [13,20,24]. The RAD50 binding site (RBS; aa residues 429–461) is localized between the DBD, and interact with the coiled-coil domain of RAD50. The conserved glycine-arginine-rich motif (GAR motif; amino acid residues 566–600) is methylated by protein arginine methyltransferase 1 (PRMT1), at the MRE11 residues Arg570, Arg572, Arg574, Arg576, Arg577, Arg580 [25,26]. This methylation allows sensitive regulation of MRE11 nuclease activity but does not affect the MRN complex assembly.

The MRE11 protein forms a binding clamp that tethers RAD50 and NBN, which otherwise cannot interact directly [1]. For the MRN complex assembly, each MRE11 molecule first binds to a single RAD50 protein. Subsequently, the MRE11-RAD50 dimer homodimerizes to form a MRE11_2_RAD50_2_ core, bridged and interlocked by two NBN subunits (Figure 1) [15,27].

### 2.2. The RAD50 ATPase

The *RAD50* gene, located on chromosome 5q31.1, encodes for a primary transcript consisting of 25 exons (NM_005732). Its protein product, the RAD50 ATPase (OMIM: *604040) consists of 1312 amino acids (with a molecular mass of 150 kDa) and forms the largest component of the MRN complex.

**Figure 3:**
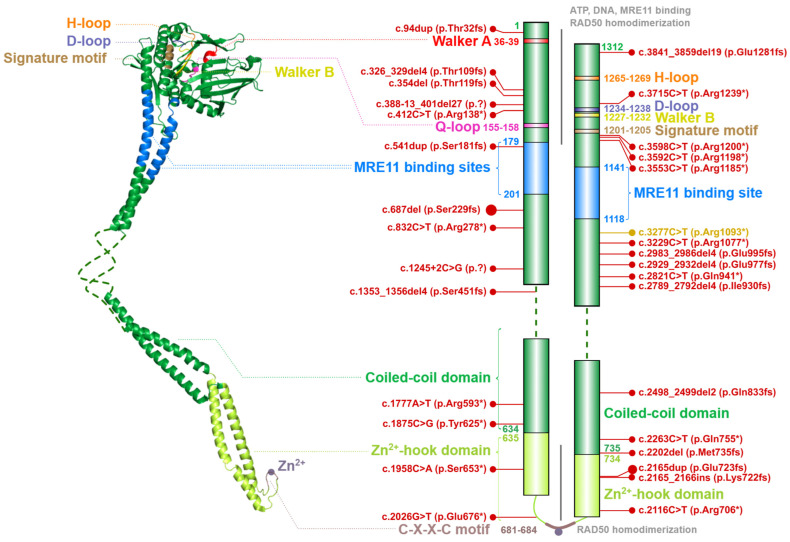
Schematic structure of the RAD50 monomer (left) modelled from globular part of *Ch. thermophilum* protein (PDB ID: 5DAC) and human coiled-coil domain (PDB ID: 5GOX). Dashed line denotes the unstructured non-crystallized parts. Colors of protein domains in structure model (left) correspond to that in schematic bar chart that summarize the domain composition and the most frequent germline loss-of-function variants with allele frequency >10^−5^ in the GnomAD database (in red, the size of the lollipops reflects the frequency of a variant). Yellow lollipop corresponds to NBSLD-causing variant. Grey notes highlight important domain interactions.

The entire RAD50 polypeptide chain is folded in half to form a fibrillar structure with antiparallel helices that have a Zn^2+^-hook on one side (formed by the central portion of the polypeptide) and a globular head containing the catalytic ATPase domain (assembled from N- and C-terminal ends of the polypeptide) on the other one (Figure 3). A similar structural assembly is found in other RAD50 homologs belonging to the structural maintenance of chromosomes (SMC) protein family [28].

The ATPase domain consists of six conserved structures, including the Walker A (WA; aa residues 36–39) and Walker B (WB; aa residues 1227–1232), Q-loop (Q-L; aa residues 155–158), signature motif (SM; aa residues 1201–1205), D-loop (D-L; aa residues 1234–1238) and H-loop (H-L; aa residues 1265–1269). These conserved nucleotide-binding motifs are required for interactions with ATP and MRE11 and for the DNA binding [7,20,21,29,30].

A flexible arm, forming an antiparallel coiled-coil domain localized between MRE11 binding sites, is tipped by a hook domain containing a conserved CXXC motif that includes two invariant cysteine residues (Cys681 and Cys684) separated by two hydrophobic amino acids (X). The CXXC motif binds a Zn^2+^ ion and forms the hook domain mediating RAD50 homodimerization controlled by the ATP binding [13,31,32]. The homodimerization by Zn^2+^-hook domain include two RAD50 molecules from the same MRN complex and form a predominantly ring-shaped form of MRN complex (Figure 1). However, the interaction of Zn^2+^-hook domains may also form an intercomplex that is required for long-range tethering of two DNA molecules, such as the resected DNA and its sister chromatid during homologous recombination [7,33,34].

The ATP-dependent RAD50 conformation changes regulate the nuclease activity of MRE11. Once ATP is bound within the globular head of each RAD50 monomer, the RAD50 molecules reach a rigid ‘closed’ conformation and their head domains interact with each other to form a groove appropriate for the accommodation of dsDNA [9,35]. In the ATP-bound, closed state, RAD50 blocks access of dsDNA to MRE11 active site and prevents its nuclease activity. After ATP hydrolysis which leads to large conformational change of RAD50, dsDNA is accessible to the nuclease cleavage by MRE11 [36,37]. Thus, hydrolysis of ATP by RAD50 renders RAD50-MRE11 dimer to the ‘open’ conformation with high processivity of MRE11 exonuclease and endonuclease activities.

### 2.3. NBN, a Dynamic Connector

The *NBN* gene, localized on chromosome 5q31.1, codes for a canonical transcript consisting of 16 exons (NM_002485). Its protein product, NBN protein (Nijmegen breakage syndrome protein 1; OMIM: *602667; also known as nibrin or NBS1) consists of 754 amino acids (with molecular mass 85 kDa) [38]. The NBN protein acts as a phosphoprotein-binding and adapter subunit of eukaryotic MRN complexes providing the MRE11_2_RAD50_2_ tetramer with a versatile connector to various signaling or DSB repair proteins [20].

**Figure 4:**
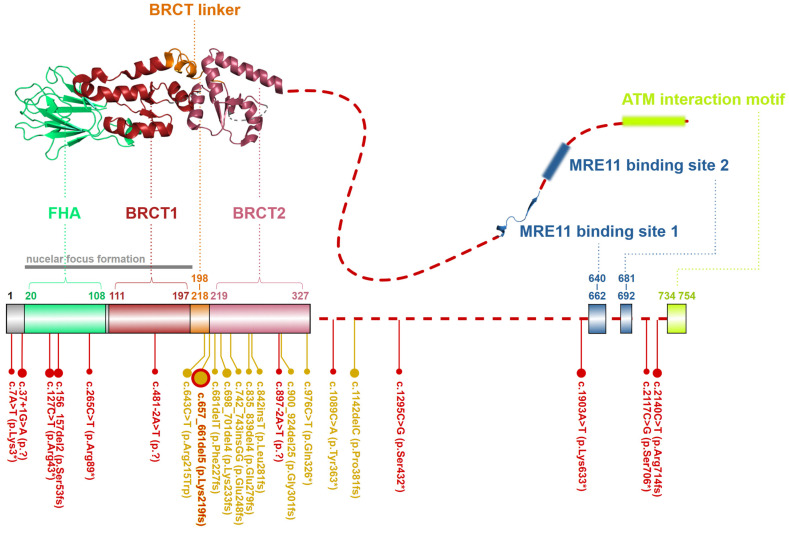
Schematic structure of the NBN protein from *S. pombe* (up, PDB ID: 3HUE). A dashed line denotes the unstructured non-crystallized parts. Colors of protein domains in structure model (up) correspond to that in schematic bar chart summarizing domain positions and the most frequent germline loss-of-function variants with allele frequency >10^−5^ in the GnomAD database (the size of the lollipops reflects the frequency of a variant). Yellow lollipops correspond to NBS-causing variants. Grey notes highlight the important domain interaction.

Its N-terminal part contains a forkhead-associated domain (FHA; aa residues 20–108) and two BRCA1 C-terminal domains (BRCT1; aa residues 111–197 and BRCT2; aa residues 219–327) separated by the BRCT linker (BRCTL; aa residues 198–218; Figure 4). FHA and BRCT domains bind multiple phosphorylated proteins regulating the MRN complex interactions. Through an FHA domain, NBN binds the C-terminal-binding protein interacting protein (CtIP; aka retinoblastoma-binding protein 8-RBBP8) [39]. FHA together with BRCT1/2 domains interacts with a phosphorylated mediator of the DNA damage checkpoint 1 (MDC1), which promotes recruitment of repair proteins to the sites of DNA breaks [40,41]. The rest of NBN polypeptide represents a largely unstructured region except for two MRE11 binding sites (MBS1; aa residues 640–662 and MBS2; aa residues 681–692) and ATM interaction motif (AIM; aa residues 734–754), through which NBN recruits ATM to the proximity of DSB. In turn, ATM phosphorylates multiple proteins at chromatin including γ-H2AX (H2A histone family member X) and MRN complex itself, including Ser278 and Ser343 residues of NBN [9,13,31,42].

Folding of the MRN complex in the cytosol is facilitated by a chaperon R2TP complex (consisting of PIH1 domain-containing protein 1 (PIH1D1), RNA polymerase II-associated protein 3 (RPAP3) and RUVB-like AAA ATPase 1 and 2 (RUVBL1 and RUVBL2)) [43]. Subsequently, the nuclear localization signal of NBN promotes translocation of the MRN to the nucleus [44]. By direct binding to MRE11, NBN stabilizes the MRN complex and stimulates the MRE11 nuclease activity [43]. Moreover, MRE11-NBN interaction is required for genome integrity and tumor suppression [45]. An important interacting partner of NBN is CtIP protein, a fourth eukaryotic, non-catalytic MRN complex subunit, which is essential for the initiation of DNA ends resection. The CtIP is retained at the break site by phosphorylation-dependent binding to the FHA and BRCT domains of the NBN [27,46,47].

While MRE11 and RAD50 homologs and their M_2_R_2_ complexes are ubiquitous across living organisms, the NBN protein (or its homolog XRS2 in *Saccharomyces cerevisiae*) is a characteristic component of eukaryotic cells only. Recent cryoelectron microscopy analysis of the eukaryotic MRN complex from *Ch. thermophilum* by Rotheneder and colleagues described a global architecture, revealing a rod-like assembly of RAD50 dimers protruding with their CC domains 60 nm apart from a complex of RAD50 globular head with MRE11 homodimer stabilized by an asymmetrically-bound single NBN molecule [14]. Despite that this work shed light on the possible composition of the human MRN complex, many important questions remained unanswered, including its dynamics during the DSB repair or the composition of the NBN subunit and its interaction with binding partners, including ATM or CtIP [48].

## 3. The MRN Complex Function in DSB Repair

**Figure 5:**
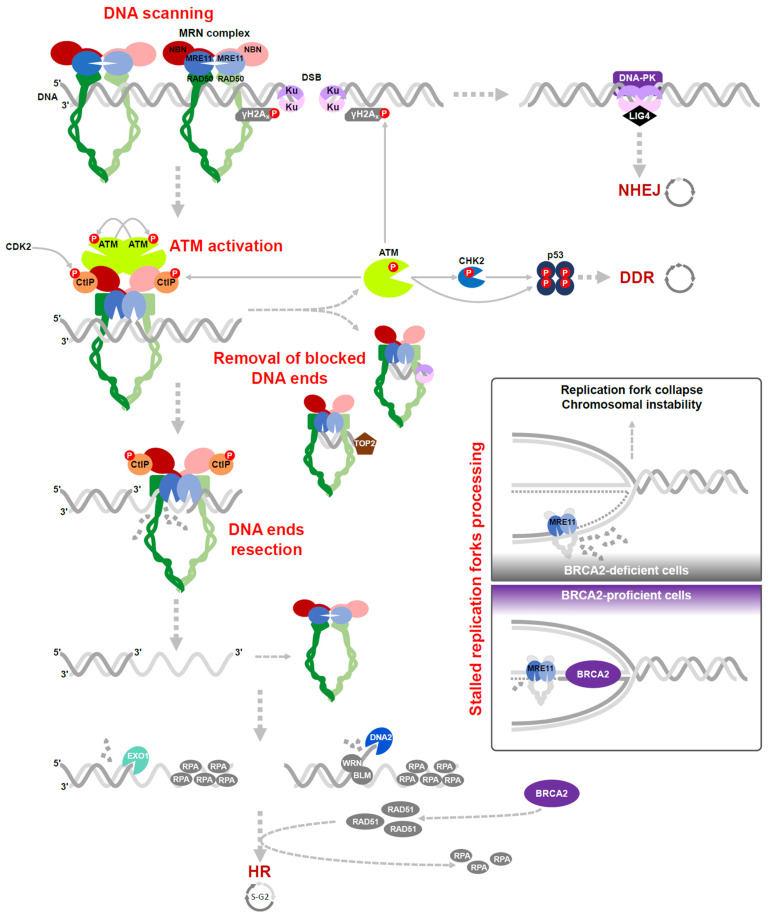
Schematic diagram representing the crucial roles of the MRN complex in sensing and processing blocked DNA ends and in the processing of stalled replication forks. Additional details are provided in the text. BLM–Bloom syndrome protein; BRCA2—breast cancer type 2 susceptibility protein; CDK2—cyclin-dependent kinase 2; CHK2—checkpoint kinase 2; DNA2—DNA replication helicase 2; DNA-PK—DNA protein kinase; EXO1—exonuclease 1, LIG4—ligase 4; p53—tumor suppressor protein p53; RAD51—DNA repair protein RAD51 homolog 1; RPA—replication protein A; TOP2—DNA topoisomerase II; WRN—Werner syndrome helicase.

The MRN complex is a crucial part of a network sensing DSB and initiating DDR. The MRN complex assembly contributes to the formation of DNA repair foci surrounding the DNA-damaged sites, as indicated by the presence of γ-H2AX phosphorylated at the Ser139 by ATM and located up to 2-Mbp distances from the DSB [7,49]. Although many details remain to be elucidated, the last two decades have allowed to assign the key functions of the MRN complex in the DSB repair (Figure 5).

### 3.1. ATM Activation

An important activity of the MRN complex is the recruitment of ATM through its interaction with the C-terminal motif of NBN (Figure 5). Following DNA damage, ATM undergoes autophosphorylation at Ser1981, leading to dissociation of the inactive homomultimeric ATM complex into active ATM monomers [50]. In turn, the ATM kinase phosphorylates numerous targets, including histones and proteins involved in HR repair [51]. ATM activity phosphorylates both the chromatin components involved in DNA repair (including histone H2AX, CtIP, EXO1, RPA, and many others) and the cell cycle regulators (including CHK2 and tumor suppressor p53) involved in the temporal checkpoint arrest. Thus, loss of the MRN complex in the hereditary syndromes (ATLD and NBS) is associated not only with impaired DNA repair, but also with defective checkpoint that manifests as radioresistant DNA synthesis [52].

### 3.2. Removal of Blocked DNA Ends

Broken DNA ends rapidly associate with Ku proteins due to their high abundance in nuclei of human cells [53]. The MRN complexes have the capacity to scan dsDNA (Figure 5) bound to nucleosomes in the vicinity of DSB via the ATPase domain of RAD50. Ku proteins associated with broken DNA ends recruit the DNA-dependent protein kinase (DNA-PK) that stimulates recruitment of the ligase IV complex, providing a DNA re-ligation in canonical NHEJ repair. Homology-directed repair requires large 3′-ssDNA overhangs free of any protein adducts. Thus, Ku proteins and DNA-PK must be detached from DNA ends in order to activate HR [54]. Ku proteins or other protein adducts (including the covalently bound topoisomerase II; Figure 5) are released by endonuclease cleavage provided by MRE11 [55]. Activation of MRE11 endonuclease activity requires topological change of the MRN complex induced by ATP hydrolysis in RAD50 [20]. Moreover, the MRE11 endonuclease activity is stimulated by phosphorylated CtIP, an MRN cofactor, interacting with the complex via FHA domain in the NBN protein [46]. Activity of CtIP is enhanced by CDK2 phosphorylation in S-G2 phase of the cell cycle but CtIP is ubiquitinated at the end of mitosis and targeted to proteasomal degradation [56]. Thus, the absence of CtIP prior to S phase favors NHEJ over HR at the beginning of the cell cycle when sister chromatid is not available for homologous-directed repair. Therefore, CtIP contributes to the selection between NHEJ and HR.

### 3.3. Resection of DNA Ends

After nicking DNA molecule, the MRN complex proceed a short-range degradation of a DNA strand toward its 5′ terminus by enhancing its 3′-5′ exonuclease activity [57]. This initial DNA trimming makes room for subsequent long-range DNA resection, that is already independent on the MRN complex activity. This resection is performed in 5′-3′ direction by exonucleases EXO1 or DNA2 in complex with BLM and WRN helicases (Figure 5). In turn, the formed 3′-ssDNA overhangs are covered by RPA proteins and subsequently, they are exchanged for the RAD51 recombinase by the activity of BRCA2 complex [1,58]. Loading of RAD51 promotes a strand invasion, which enable a search for homologous sequence in sister chromatid that is required for template-directed reconstruction of the missing DNA sequence [42,58,59].

### 3.4. Processing of the Stalled Replication Forks

Besides its established function in DSB repair, there is emerging evidence for an involvement of the MRN complex in DNA replication. In particular, MRN has recently been implicated in the remodeling of the stalled replication forks (Figure 5) promoting the replication restart [9]. Recruitment of MRE11 to the stalled forks depends on functional p53, whereas the mutant p53 promotes processing of the forks by mutagenic DNA repair pathways [60]. On the other hand, excessive nuclease activity of MRE11 can lead to degradation of the stalled replication forks in BRCA2-deficient tumors [61]. Thus, MRN function during replication is controlled by two major tumor suppressor pathways, BRCA2 and p53, and may also modulate the response of cancer cells to chemotherapy.

In summary, the proteins of the MRN complex serve as a signaling hub that controls the choice for DSB repair depending not only on the nature of the DNA damage but also on the cell cycle context. For a detailed description of the mechanisms controlling the DSB repair we refer the reader to several excellent recent reviews [62,63,64]. An important part of the MRN complex activity involves activation of the ATM kinase, as evidenced by the phenotypic characteristics of individuals carrying germline inactivation of both alleles in ATM or MRN complex genes, which share a number of syndromic similarities.

## 4. Germline Alterations of MRN Complex Genes in Autosomal Recessive Syndromes

Biallelic inactivation of *MRE11*, *RAD50*, or *NBN* lead to the rare autosomal recessive (AR) syndromes of chromosomal instability, with partially overlapping clinical characteristics in which dominate neurological symptoms and susceptibility to malignancies (Table 1). Severe clinical manifestation of biallelic MRN gene defects arise from impaired MRN complex assembly and function; however, the development of neurodegenerative or neurodevelopmental pathologies (progressive cerebellar degeneration or microcephaly, respectively) are poorly understood [65]. Syndromes caused by MRN genes impairment share some typical features with other chromosome instability syndromes caused by biallelic inactivation in the gene(s) coding for proteins that functionally interact with the MRN complex.

### 4.1. The Nijmegen Breakage Syndrome (NBS)

The Nijmegen breakage syndrome (NBS; also known as Seemanova syndrome, OMIM: **#**251260) caused by biallelic germline inactivation of the *NBN* gene is the most common syndrome associated with the biallelic inactivation of proteins involved in the assembly of the MRN complex. The population frequency of truncating and nonsense *NBN* variants in the GnomAD database ranges from 0.03% in Latino/Admixed Americans to 0.1% in European–Finnish). However, more than 90% of NBS patients come from Slavic Eastern European populations (underrepresented in GnomAD) and are typically homozygous for the Slavic pathogenic founder deletion c.657_661delACAAA (mostly referred to as c.657del5) in exon 6 [66]. Ten additional truncating rare NBS-causing variants have been identified (Figure 4). These rare variants are located between exons 6 and 10 and are predicted to truncate NBN protein [67,68]. The study by Seemanova et al. reported that the c.657del5 could be found in all Slavic populations but a particularly high frequency of heterozygotes (0.5–1.0%) can be found in populations of Slavs from the Czech Republic, Poland, Ukraine, Bulgaria, and in Sorbs in Germany [69]. Although the c.657del5 deletion was originally considered as a null mutation, it was later reclassified as a hypomorphic variant with only a partial loss of the NBN function [39,66,67]. The deletion causes a frame-shift resulting in formation of the N-terminal 26 kDa fragment truncated at the beginning of the BRCT2 domain (Figure 4), and the C-terminal 70 kDa NBN protein fragment generated from an alternative translation start localized upstream from the deletion [69,70]. Experimental data in mouse models show that all biallelic *NBN* null mutations are lethal, however, the 70 kDa isoform of NBN is a hypomorph retaining residual survival-promoting activity [71]. It is assumed that the survival of NBS patients is promoted by the presence of protein-protein interactions at the C-terminus, whereas malignancy and immunodeficiency can be attributed to the absence of the protein-protein interactions at the N-terminus, which is deleted in p70-nibrin [70,71]. It was found that some NBS patients have milder phenotype due to an alternative mRNA splicing [67]. The insertion c.742_743insGG in exon 7 is an example of such alteration in the *NBN* gene, creating a new alternative splice site and leads to the excision of exons 6–7 from the mRNA. Subsequently, spliced mRNA is translated to 80 kDa protein containing both C-terminal and N-terminal interaction domains. Given that the c.657del5 founder mutation is localized in exon 6, these findings have important implications for the potential treatment of the NBS patients based on the directed alternative splicing to remove exon 6 and exon 7 from the *NBN* [70].

The symptoms of NBS (Table 1) include mental and growth retardation with congenital microcephaly, chromosome instability, immunodeficiency, radiosensitivity and increased risk of lymphoid tumors (dominantly non-Hodgkin lymphoma). The typical craniofacial features include receding forehead and mandible and prominent mild face with long nose [65]. All female NBS patients are infertile (data are limited for males) and germline variants in NBN should be considered as a rare cause of infertility [72,73]. The median age at cancer onset was 9.1 years (with interquartile range 5.9–14.0 years) and the probability of 20-year survival was 44.6%, as reviewed in a cohort of 241 NBS patients from 11 countries by Wolska-Kusnierz and colleagues in 2020 [74]. There is no specific treatment for NBS patients currently; however, the hematopoietic stem cell transplantation extends the life expectancy in NBS patients, preventing both immunodeficiency and malignancy [74]. Severe hypersensitivity to standard chemotherapy was observed in 17 NBS patients treated for non-Hodgkin lymphoma with reduced doses of standard chemotherapy to 80%, all of whom experienced grade 4 toxicity and two of whom died from treatment-related complications [75]. Moreover, radiotherapy must be entirely excluded due to an extreme radiosensitivity that can be fatal in NBS patients [76]. Therefore, an early diagnosis is necessary for appropriate preventive care, which primarily includes avoiding cancer risk factors [77,78,79]. An appropriate surveillance management was suggested for the relatives of NBS patients [68,69,72,80].

### 4.2. Ataxia-Telangiectasia-like Disorder (ATLD)

Biallelic germline inactivation of the *MRE11* gene results in an autosomal recessive Ataxia-telangiectasia-like disorder (ATLD1; OMIM: **#**604391) that, together with ataxia-telangiectasia (AT; OMIM: #208900; caused by *ATM* inactivation), belongs to spinocerebellar ataxias characterized by disturbances of eye movement or oculomotor apraxia and DNA damage hypersensitivity [79,81,82,83] (Table 1). The population frequency of germline loss of function variants in *MRE11* ranges from 0.009% in European–Finnish to 0.1% in East Asian in GnomAD database; however, ATLD is an extremely rare syndrome. Mahale and colleagues reported 23 individuals with ATLD identified until 2020 [84]. A few ATLD-causing variants in *MRE11* have been reported (Figure 2), resulting in lower levels of MRE11 protein or inability to interact with its protein partners [79,85]. The clinical presentation of ATLD overlaps with AT and NBS (radiosensitivity and chromosomal instability), ATLD and AT show neurodegeneration, whereas NBS is characterized by microcephaly [86]. Compared with AT, symptoms of ATLD have a later onset, slower progression and milder phenotypes [87]. However, individual cases of ATLD may develop different phenotypes [88]. Current evidence does not suggest that ATLD patients develop myeloid tumors, as only two patients who died from a cancer diagnosis have been described (two brothers who developed lung cancer in childhood) [89]. Therefore, the contribution of ATLD to cancer predisposition remains unknown. An X-ray exposure and radiotherapy should be avoided in patients with ATLD [87].

**Table 1:** Phenotype characteristics of inherited syndromes caused by biallelic germline pathogenic variants in the genes coding for the MRN complex proteins.

		Nijmegen Breakage Syndrome (NBS)	NBS-like Disease (NBSLD)	Ataxia Telangiectasia-like Disease (ATLD)
Gene		*NBN*	*RAD50*	*MRE11*
Inheritance		AR	AR	AR
Described syndromic individuals		>1000	2	~30
Common features	Chromosomal instability	Yes	Yes	Yes
	Ionizing radiation hypersensitivity	Yes	Yes	Yes
	Intellectual disability	Mild- moderate	Yes	Variable (limited evidence)
Less common features	Microcephaly	Yes	Yes	No/Yes *
	Short stature	Yes	Yes	No
	Craniofacial dysmorphism	Yes	Yes	No
Unique features	Immunodeficiency	Yes	No	No
	Increased rick (especially lymphoid tumors)	Yes	No	No
	Cerebellar ataxia/oculomotor apraxia	No	No	Yes
Other features	Telangiectasia	No	No	No
	AFP level	Normal	Normal	Normal

* presented in two unrelated patients [88].

### 4.3. Nijmegen Breakage Syndrome-like Disorder (NBSLD)

Germline biallelic pathogenic variants of *RAD50* have been shown to cause autosomal recessive Nijmegen breakage syndrome-like disorder (NBSLD; OMIM: **#**613078). This disorder is associated with an increased risk of malignancies [79,82]. The highest population frequency of loss-of-function variants in *RAD50* ranges from 0.06% (in Ashkenazi Jewish) to 0.3% (in European–Finnish) in the GnomAD database; however, only two NBSLD patients have been described so far [82]. Biallelic variants (marked in Figure 3) in *RAD50* show clinical features similar to both NBS and ATLD [79,90]. NBSLD is characterized by radioresistant DNA synthesis with radiation hypersensitivity and neurodegeneration but no immunodeficiency [79,82,90].

## 5. Heterozygous Germline Alterations of MRN Complex Genes in Cancer Predisposition

Germline pathogenic variants in the MRN complex genes in heterozygous state have been associated with an increased cancer risk in a broad range of diagnoses. However, the frequency of alterations in the MRN complex genes is approximately 1% or less in cancer patients and the rarity of variant carriers in majority of the studies, the lack of international consortia effort, and the insufficient meta-analyses hamper reliable estimation of the risk, associating with germline alterations in individual MRN genes.

### 5.1. Heterozygous Germline Variants in NBN

Carriers of heterozygous alterations in the *NBN* gene are most common in Europeans of Slavic origin due to the high prevalence of founder c.657del5 variant, discussed above. In contrast to infertile NBS female patients, females heterozygous for c.657del5 have normal or even increased reproductive fitness [69]. However, this variant in the heterozygous state has been associated with a moderately-increased susceptibility to various cancers. Numerous studies (mostly from the Central European region) analyzed an association of c.657del5 with the risk of colorectal, pancreatic, prostate, ovarian, and breast cancer (BC) or brain tumors (Table 2).

The majority of *NBN* studies originate from Slavic European populations and analyzed female breast cancer patients, but they consistently failed to find an association with increased breast cancer risk for heterozygotes with *NBN* germline alterations. Furthermore, this lack of association between *NBN* germline variants and breast cancer risk was supported by the negative results of the two largest analyses examining germline variants in cancer susceptibility genes in female breast cancer patients [91,92]. In contrast, some studies have reported an increased risk of lymphoid tumors, melanoma, ovarian, pancreatic and prostate cancer (Table 2). A recent meta-analysis of studies in prostate cancer patients confirmed an association between the *NBN* germline alterations and increased prostate cancer risk (with OR = 6.4 and OR = 7.5 for the total and Caucasian populations, respectively) [93]. Episodic reports have associated germline *NBN* variants with the risk of cervical [94] and hepatocellular carcinoma [95], medulloblastoma [96], or hematopoietic malignancies [94,97]. Interestingly, recent analysis of 34,046 US patients by Belhadj et al. confirmed the lack of association with BC, but suggests a potential role of *NBN* germline pathogenic variants in the development of other cancer types [98].

Taken together, heterozygous germline pathogenic alterations in the *NBN* gene probably do not predispose female carriers to breast cancer, but significantly increase the prostate cancer risk in male carriers. However, a convincing identification of the cancer risk spectrum associated with germline *NBN* variants is still lacking and the large studies/meta-analyses including the populations with increased prevalence of germline *NBN* variants will be of high importance.

### 5.2. Heterozygous Germline Variants in MRE11 and RAD50

Even more than for *NBN*, the clinical significance of heterozygous germline alterations in the *MRE11* and *RAD50* genes remains elusive. Analyses of the *RAD50* gene have shown that its germline variants are associated with colorectal [99], pancreatic [100], hepatocellular [95] or breast cancer risk [101,102,103]. The recurrent, loss-of-function, germline, Finnish founder variant c.687delT (p.Leu229Ter) has been associated with increased breast cancer risk (OR = 4.3; 95% CI 1.5–12.5) in the Finnish population [101]. However, this association has not been confirmed in non-Finnish European populations [104]. Recent large studies of female breast cancer patients failed to find association of the germline pathogenic (truncating) variants in *MRE11* and *RAD50* with breast cancer [91,92].

**Table 2:** Representative studies analyzing associations of germline *NBN* variants with cancer risk. Shown studies identified more than single carrier of any *NBN* truncating variant in patients and analyzed the frequency of *NBN* germline variants in controls. (Significant associations highlighted in bold).

Malignancy	Country	Patients (%) *	Controls (%) *	OR (95% CI); *p*-Value **	Ref.
Brain	**PL**	**3/104 (2.9)**	**74/12484 (0.6)**	**4.9 (4.4–5.3); 0.003**	**Ciara 2010 [96]**
	**PL**	**6/102 (5.9)**	**0/300 (0)**	**40.5 (2.3–721.2); <0.001**	**Trubicka 2017 [105]**
Breast	PL	5/230 (2.2)	3/530 (0.6)	3.9 (0.9–16.4); 0.06	Gorski 2003 [106]
	PL	17/2012 (0.8)	18/4000 (0.5)	1.9 (1.0–3.7); 0.09	Gorski 2006 [106]
	PL	2/181 (1.1)	21/4000 (0.5)	2.1 (0.5–9.1); 0.6	Kanka 2007 [107]
	PL	4/224 (1.8)	10/1620 (0.6)	2.9 (0.9–9.4); 0.08	Steffen 2004 [94]
	PL	2/270 (0.7)	2/295 (0.7)	1.1 (0.2–7.9); 1.0	Roznowski 2008 [108]
	US	48/28,536 (0.2)	39/26,264 (0.1)	1.1 (0.7–1.8); 0.59	Couch 2017 [109]
	DE	12/5589 (0.2)	9/2189 (0.4)	0.5 (0.2–1.2); 0.15	Hauke 2018 [110]
	PL	18/2464 (0.7)	22/4000 (0.6)	1.3 (0.7–2.5); 0.46	Rogoża-Janiszewska 2020 [111]
	US	57/32,247 (0.2)	51/32,544 (0.2)	1.1 (0.7–1.6); 0.81	Hu 2021 [91]
	CN	6/8067 (0.07)	5/13,129 (0.04)	2.0 (0.6–6.4); 0.35	Fu 2021 [112]
	US	53/26,384 (0.20)	115/64,649 (0.18)	1.3 (0.9–1.8); 0.14	Kurian 2017 [113]
	CZ	8/703 (1.1)	9/915 (1.0)	1.2 (0.5–3.0); 0.81	Mateju 2012 [114]
Colorectum	PL	3/234 (1.3)	10/1620 (0.6)	2.1 (0.6–7.7); 0.22	Steffen 2004 [94]
	CZ	3/750 (0.4)	5/1411 (0.35)	0.95 (0.2–4.2); 0.95	Pardini 2009 [115]
Lymphoid	**RU**	**2/68 (2.9)**	**0/548 (0)**	**41.2 (1.9–862.9); 0.01**	**Resnick 2003 [116]**
	**PL**	**2/42 (4.8)**	**10/1620 (0.6)**	**8.1 (1.7–37.9); 0.03**	**Steffen 2004 [94]**
Melanoma	**PL**	**4/105 (3.8)**	**10/1620 (0.6)**	**6.4 (1.9–20.7); 0.008**	**Steffen 2004 [94]**
	**CZ**	**7/264 (2.7)**	**4/1479 (0.3)**	**10.0 (2.5–47.0); <0.001**	**Stolarova 2020 [117]**
Ovarian	US	9/3257 (0.3)	8/3447 (0.2)	1.2 (0.5–3.1); 0.97	Ramus 2015 [118]
	**CZ**	**14/1320 (1.1)**	**7/2278 (0.3)**	**3.5 (1.3–10.2); 0.006**	**Lhotova 2020 [119]**
	**US**	**17/5020 (0.34)**	**115/64,649 (0.18)**	**1.85 (1.1–3.2); 0.03**	**Kurian 2017 [113]**
Pancreas	**PL**	**8/383 (2.1)**	**22/4000 (0.6)**	**3.8 (1.7–8.6); 0.002**	**Lener 2016 [120]**
	**CZ**	**5/241 (2.1)**	**2/915 (0.2)**	**9.7 (1.9–50.2); 0.006**	**Borecka 2016 [121]**
Prostate	**PL**	**9/340 (2.6)**	**9/1500 (0.6)**	**4.5 (1.7–11.5); 0.002**	**Cybulski 2004 [122]**
	US/FI/DE	5/2127 (0.2)	0/697 (0)	3.61 (0.2–65.3); 0.58	Hebbring 2006 [123]
	**PL**	**63/4162 (1.5)**	**23/3956 (0.6)**	**2.6 (1.6–4.3); <0.001**	**Cybulski 2013 [124]**
	PL	11/390 (2.8)	3/308 (0.9)	3.0 (0.8–10.7); 0.1	Wokołorczyk 2020 [125]

* carriers of germline *NBN* truncations/all analyzed individuals; ** OR–odds ratio; 95% CI–95% confidence interval. CN—China, CZ—Czech Republic, DE—Germany, FI—Finland, PL—Poland, RU—Russia, US–the USA.

The germline alterations in *MRE11* are probably rare and were reported episodically in mesothelioma patients [126] and in breast cancer patients [103,127,128]. Castéra and colleagues identified 11 carriers of pathogenic or potentially pathogenic germline alterations of MRN complex genes in 708 hereditary breast cancer patients [129]. These included four alterations in *MRE11* (three of which were protein-truncating variants) and two in *RAD50*. Recently, Elkholi and colleagues identified a stop-gain c.1516G >T (p.Glu506*) variant in *MRE11* in two unrelated French-Canadian patients from hereditary breast/ovarian cancer families [130]. However, a subsequent case-control study found no carrier of this variant in 1925 breast cancer, 341 ovarian cancer and 367 endometrial cancer patients from the same population. LaDuca et al. found no association of rare germline pathogenic variants in *MRE11* and *RAD50* with any cancer type in an analysis of 165,000 high-risk cancer patients [131]. Hu et al. analyzed 38,332 US breast cancer patients and identified 28 (0.07%) carriers of pathogenic germline variants in *MRE11*, 66 (0.17%) in *NBN*, and 72 (0.19%) in *RAD50* but these frequencies did not differ from that in controls [91]. Similar frequencies of germline pathogenic variants in MRN complex genes (0.10%, 0.18%, and 0.25%, respectively) and lack of association with the breast cancer were found in a parallel study of 48,826 breast cancer patients by the BCAC consortium (The Breast Cancer Association Consortium) [92].

In conclusion, the most common germline, heterozygous alterations found in cancer patients affect *NBN* in Slavic European populations, and occur less frequently in other populations worldwide with a frequency similar to *RAD50* variants (~0.2% cancer patients). Germline variants in *MRE11* are rarely identified (<0.1% of cancer patients). The associations between germline alterations in MRN complex genes and predisposition to specific cancer types in terms of Mendelian inheritance remains poorly understood. However, co-occurring germline variants in MRN genes may modify cancer risk in carriers of multiple germline pathogenic variants in cancer predisposition genes [132]. Recent observations suggest that germline pathogenic variants in the MRN complex genes are unlikely to predispose to breast cancer and therefore the breast cancer-specific surveillance should not be offered to female carriers of germline pathogenic variants affecting the MRN complex genes. On the other hand, the association with other cancers remains to be established. Although there are currently no specific preventive recommendations for carriers of germline pathogenic variants in the MRN complex genes, this may change when meta-analyses or analyses in unselected cancer populations (e.g., carriers of NBN germline alterations with prostate cancer) are performed. In addition, the presence of germline alterations in MRN complex genes has potential predictive value for targeted anticancer therapy, as discussed below.

## 6. Somatic Alterations in MRN Complex Genes in Tumors

Somatic and germline alterations in the HR repair genes may represent an important predictive information guiding the anticancer treatment with platinum compounds [133] or poly-(ADP-ribose)-polymerase inhibitors (PARPi) [134]. The PARPi studies were initially focused on ovarian cancer patients because about 25% of them carry a germline alteration in *BRCA1* or *BRCA2* [119,135]. An increasing popularity of a tumor-agnostic approach has expanded the indication of various PARPi to a much broader spectrum of tumor types characterized by the presence of defects in genes encoding HR repair, including the genes encoding the MRN complex. Beyond patients with ovarian tumors, the most flexible indication concerning the defects in HR repair genes concerns patients with prostate cancer. In TALAPRO-1 phase 2 trial of talazoparib, a stable disease was achieved in patients with metastatic castration-resistant prostate cancers carrying the alterations in rarer HR repair genes including *MRE11* and *NBN* [136]. A combined therapy with olaparib and double immunotherapy (durvalumab and tremelimumab) showed its efficacy in patients with alterations in HR repair genes (including *NBN*, *RAD50* and *MRE11*) in breast, ovarian, pancreatic, endometrial, or prostate cancers [137]. The repository of clinical trials (https://clinicaltrials.gov) currently (assessed on 24 January 2023) registers more than 20 studies validating the efficacy of PARPi in monotherapy or in combination in patients with alterations in MRN genes and various tumor types, including breast, biliary, gastric, lung, ovarian, pancreatic, or prostate cancer, melanoma, and sarcoma.

The somatic inactivating alterations (indels and nonsense mutations) in MRN genes are infrequent (less than 1% of analyzed samples) according to the COSMIC (Catalogue of Somatic Mutations in Cancer) database (https://cancer.sanger.ac.uk/cosmic; accessed on 26 November 2022). However, these defects systematically occur in endometrial, gastrointestinal, skin tumors. The frequency of somatic missense variants is higher and in particular cancers (incl. prostate, endometrium, ovary) reach up to 5% of all COSMIC database samples [138]. Interestingly, some alterations were demonstrated to predict treatment response. Al-Ahmadie et al. identified a hemizygous, somatic p.Leu1237Phe variant in *RAD50* using a WGS (whole genome sequencing) in tumor sample in a patient with metastatic small-cell cancer of the ureter [139]. The authors concluded that this variant, affecting the D-loop RAD50 motif, was the likeliest contributor to the complete response to systematic combinational therapy by AZD7762 (an ATP-competitive checkpoint CHK1/2 kinase inhibitor blocking the ATR/CHK1 DNA repair pathway) and irinotecan (a topoisomerase I inhibitor). The authors performed phenotypical characterization of p.Leu1237Phe mutants in yeast and mouse cell models and demonstrated that p.Leu1237Phe is a hypomorphic variant partially destabilizing D-loop RAD50 structure required for a proper ATM activation. The biochemical consequences of the p.Leu1237Phe alteration were examined by Boswell and colleagues in a *Pyrococcus furiosus* model demonstrating that this variant impairs the D-loop-Walker A interaction influencing a rate of ATP hydrolysis and thus affecting the RAD50 regulation [140]. Seborova and colleagues identified hypermethylation of *RAD50* promoter positively correlating with platinum sensitivity in ovarian cancer patients [141]. Correspondingly, the *RAD50* hypermethylation predicted more prolonged overall survival.

The amplification of the *NBN* gene is by far the most common type of somatic alteration reported in the COSMIC database. This observation was reported by Chae and colleagues who noticed that *NBN* scored as the fourth most frequently amplified DNA repair gene displaying CNV (copy number variant) gain [142]. The analysis of 10,489 tumors performed by Wu and colleagues revealed that *NBN* amplification was the most prominent DDR gene event that occurred in over 40% of patients across 16 cancer types [143]. Moreover, the *NBN* amplification correlated with poor overall survival in ovarian patients [hazard ratio (HR) = 1.36, 95% CI 1.13 to 1.64, *p* = 9.62 × 10^−4^] and their in vitro experiments demonstrated that *NBN* amplification induced the cisplatin and PARPi resistance in breast and ovarian cancer cell lines through activation of HR pathway. Negative impact of *NBN* gain on prognosis was reported also in prostate cancer patients in univariate (HR = 3.35; 95% CI 1.6–7.01) and multivariate (HR = 3.28, 95% CI 1.56–6.89) analyses [144].

These rather episodical reports of somatic alterations affecting the MRN complex genes in cancer patients indicate that they may represent important prognostic factors but also valuable predictive biomarkers for genotoxic chemotherapy in some patients. The importance of somatic alterations in the MRN complex genes for malignant transformation processes is demonstrated by the recently reported systematic evaluation of clonal hematopoiesis in 482,789 blood-derived DNA samples by Loh and colleagues. They identified *NBN*, *MRE11*, and *ATM* among the top most frequently somatically altered genes in UK biobank patients [145].

In conclusion, the last 20 years have significantly improved our understanding of the proteins coded by the MRN complex genes, their biological functions, regulations and importance in the development of human pathologies. However, concerning the full complexity of these processes, our understanding is still halfway. While we have recognized the major structural components of the MRN complex and its dynamics, we still lack sufficient knowledge about the importance of all individual amino acids and their changes in *RAD50*, *MRE11* and *NBN* protein structures, which are ultimately required for the functional classification of the germline and somatic alterations identified in MRN complex genes. This classification can significantly improve the clinical utility of MRN gene analyses and represents a major step towards personalized management of pathological conditions associated with MRN complex alterations.

## Data Availability

All data are available within the article.
